# Human-to-monkey transfer learning identifies the frontal white matter as a key determinant for predicting monkey brain age

**DOI:** 10.3389/fnagi.2023.1249415

**Published:** 2023-11-01

**Authors:** Sheng He, Yi Guan, Chia Hsin Cheng, Tara L. Moore, Jennifer I. Luebke, Ronald J. Killiany, Douglas L. Rosene, Bang-Bon Koo, Yangming Ou

**Affiliations:** ^1^Harvard Medical School, Boston Children's Hospital, Boston, MA, United States; ^2^Department of Anatomy & Neurobiology, Boston University Chobanian and Avedisian School of Medicine, Boston, MA, United States

**Keywords:** deep learning models, human brain age estimation, monkey brain age estimation, transfer learning, brain MRIs

## Abstract

The application of artificial intelligence (AI) to summarize a whole-brain magnetic resonance image (MRI) into an effective “brain age” metric can provide a holistic, individualized, and objective view of how the brain interacts with various factors (e.g., genetics and lifestyle) during aging. Brain age predictions using deep learning (DL) have been widely used to quantify the developmental status of human brains, but their wider application to serve biomedical purposes is under criticism for requiring large samples and complicated interpretability. Animal models, i.e., rhesus monkeys, have offered a unique lens to understand the human brain - being a species in which aging patterns are similar, for which environmental and lifestyle factors are more readily controlled. However, applying DL methods in animal models suffers from data insufficiency as the availability of animal brain MRIs is limited compared to many thousands of human MRIs. We showed that transfer learning can mitigate the sample size problem, where transferring the pre-trained AI models from 8,859 human brain MRIs improved monkey brain age estimation accuracy and stability. The highest accuracy and stability occurred when transferring the 3D ResNet [mean absolute error (MAE) = 1.83 years] and the 2D global-local transformer (MAE = 1.92 years) models. Our models identified the frontal white matter as the most important feature for monkey brain age predictions, which is consistent with previous histological findings. This first DL-based, anatomically interpretable, and adaptive brain age estimator could broaden the application of AI techniques to various animal or disease samples and widen opportunities for research in non-human primate brains across the lifespan.

## Introduction

The advances of artificial intelligence (AI)-based predictor of phenotypic brain aging have generated new tools for mapping normative aging trajectory from large-scale neuroimaging data sets, by summarizing the whole-brain magnetic resonance imaging (MRI) into a holistic, simple, yet effective metric (Cole and Franke, 2017). Deep learning (DL) methods which are more advanced techniques of AI models were shown useful in providing reference information on healthy brain aging trajectories, disease-specific profiles, and for predicting disease progression. However, several challenges remain before the broader application of DL models to clinical settings. The models often require large sample sizes [at least 2,000 samples (Schulz et al., 2020; Abrol et al., 2021) for human brain MRIs] to minimize biased and confounded predictions, and the biological interpretability of the "brain age" measure is often complicated and not transparent (Moore et al., 2019).

The use of laboratory animals offers a way to run tests and experiments before human trials or when human samples (e.g., healthy brain tissues) are impossible to obtain (Gray and Barnes, 2019) and may help with improving AI models. For example, studies with rhesus monkeys (Macaca mulatta) have demonstrated how environmental and lifestyle factors such as diet or exercise impact the brain and cognition across the lifespan, and for understanding the effect of different disease pathophysiology including neurodegenerative disorders, stroke, hypertension, infectious diseases, and affect the brain as it is easier to systematically quantify and manipulate these factors in monkeys than in humans (Mellus, 1905, 1907; Roth et al., 2004; Moss et al., 2007; Lacreuse and Herndon, 2009; Mattison and Vaughan, 2017; Koo et al., 2018; Kuchan et al., 2020). However, applying AI predictors to animal models of aging (i.e., non-human primates) is rare and challenging because of the scarcity of brain MRIs (Franke et al., 2010). Contrarily, the number of publicly available human brain MRIs (>8,000) is larger than the minimum request numbers (2,000) (Schulz et al., 2020; Abrol et al., 2021) to train a DL model, yielding a good accuracy of brain age estimation on human MRIs (Feng et al., 2020; He et al., 2021a; Peng et al., 2021; Lee et al., 2022). To mitigate this issue, we implemented the transfer learning strategy, which uses knowledge learned from large-sample benchmark tasks (i.e., brain age estimation in humans) to improve prediction accuracy in small-sample target tasks (i.e., brain age estimation in animal models) (Kermany et al., 2018) in our recently developed pre-trained 3D AI models with improved transferability in 3D images (Feng et al., 2020; He et al., 2021b, 2022).

Here, we applied a DL approach to MRIs of non-human primates (NHP), which is a well-established animal model for studying cognitive aging with high genetic homology (>92%), similar cognitive and sensory abilities to those of humans, and longer lifespan (approximately 1/3 to that of human) than more distant laboratory animals such as rodents (Roth et al., 2004; Gray and Barnes, 2019). We hypothesized that five state-of-the-art 2D and 3D AI models pre-trained on 8,859 human brain MRIs could reduce the errors of AI brain age estimation in 29-290 monkey brain MRIs compared to models without transfer learning and that the key features selected by the AI models are consistent with previous biological investigations. Saliency maps generated from our models are used to examine brain image features that are most important for brain age estimation and are compared with those from our previous human brain age models. We demonstrated that 3D DL models with transfer learning are highly accurate, stable, and biologically meaningful in analyzing relatively small samples of NHPs, broadening the opportunities for applying cutting-edge AI paradigms to various laboratory animals.

## Methods

### Monkey subjects and MRI acquisition

In total, 290 T1-weighted brain MRIs from 274 rhesus monkeys were used in this study. They include 172 male and 102 female monkeys, 1–30 years of age at the time of brain MRI acquisition, 126 from the Boston University (BU) NHP database, and 148 from the PRIMatE Data Exchange (PRIME-DE) database (Milham et al., 2018).

The monkeys at BU were obtained from the Emory National Primate Research Center, the New England National Primate Research Center, and World Wide Primates for other funded projects over 3 decades. Prior to entering the study, all monkeys received medical examinations that included serum chemistry and health screenings to ensure that monkeys did not have a history of malnutrition, diabetes, chronic illness, or any neurological diseases. All monkeys were housed in the Laboratory Animal Science Center of Boston University Medical Campus, an AAALAC-accredited facility. Experiments were conducted in accordance with the Guide for the Care and Use of Laboratory Animals from the National Institute of Health's Office of Laboratory Animal Welfare and were approved by the Boston University Institutional Animal Care and Use Committee (IACUC).

MRIs on BU monkeys were obtained as previously described (Koo et al., 2012, 2013). In brief, the monkeys were anesthetized with an initial dose of ketamine (10 mg/kg, IM) followed by administration of propofol IV (25 mg/kg, IV) using a syringe pump to maintain anesthesia throughout the session or with repeated doses of ketamine (10 mg/kg, IV) and valium (1 mg/kg, IV). In the MRI scanner, monkeys were held in a stereotactic MRI-compatible head holder designed to fit within the 8-channel phase array head coil in a fixed position preventing any inadvertent movement from respiration. Respiration and oxygenation levels were monitored, and body temperature was maintained throughout the imaging session. MRI scans were performed on an Intera (for 16 scans) or Achieva 3T whole-body MRI scanner (Philips Healthcare, Best, The Netherlands). T1-weighted images were acquired using a T1-weighted 3D-turbo field echo (TFE) sequence: repetition time (TR)/echo time (TE) = 7 ms/3 ms, flip angle = 8°, NEX = 6, inter shot delay = 2,800 ms, TFE = factor 200, voxel size: = 0.6 mm isotropic, and sagittal plane acquisition.

The PRIME-DE database combines NHP neuroimaging data sets across 22 sites. Details have been described in a 2018 article (Milham et al., 2018). Of them, this study included data from 15 sites that contain *in vivo* structural MRI data for rhesus monkeys, details are described in Supplementary Table S2.

Among the 290 T1-weighted MRI scans, 260 monkeys had only one scan session, 12 had two MRI sessions, and 2 had three MRI sessions (at least 6 months apart between each scan). These longitudinal scans are treated as independent data for brain age estimation. Multiple scans from human subjects were also used in human brain age prediction studies with dozens or lower hundreds of subjects, but our study included a much lower percentage (6%) of human subjects with two scans.

### Human subjects and MRI acquisition

We collected 8,859 T1-weighted human brain MRIs (0–97 years of age, 45.2% male) from 13 public data sets, including ABIDE-I (Di Martino et al., 2014) (567 scans, age 6–57), BeijingEN^1^(180 scans, age 17–28), CamCAN (Taylor et al., 2017) (442 scans, age 18–68), DLBS (Park et al., 2012) (315 scans, age 20–89), DutchDonders^2^ (171 scans, age 18–32), IXI^3^ (556 scans, age 20–86), Narratives (Nastase et al., 2021) (335 scans, age 18–53), NIH-PD (Evans et al., 2006) (1,210 scans, age 0–23), KI-RS_Enhanced (Nooner et al., 2012) (2,032 scans, age 6–85), OASIS-3 (LaMontagne et al., 2018) (1,838 scans, age 42–97), SALD (Wei et al., 2018) (488 scans, age 19–80), Wayne (Daugherty and Raz, 2017) (606 scans, age 18–91), and YaleHires (Finn et al., 2015) (119 scans, age 18–59). These T1-weighted brain MRIs were all acquired at 1 isotropic mm voxel size or slightly higher resolution.

### Pre-processing of human and monkey MRI

Following the human brain age estimation (He et al., 2021a,b, 2022), we performed identical minimal pre-processing to both monkey and human brain MRIs.

**Figure 1:**
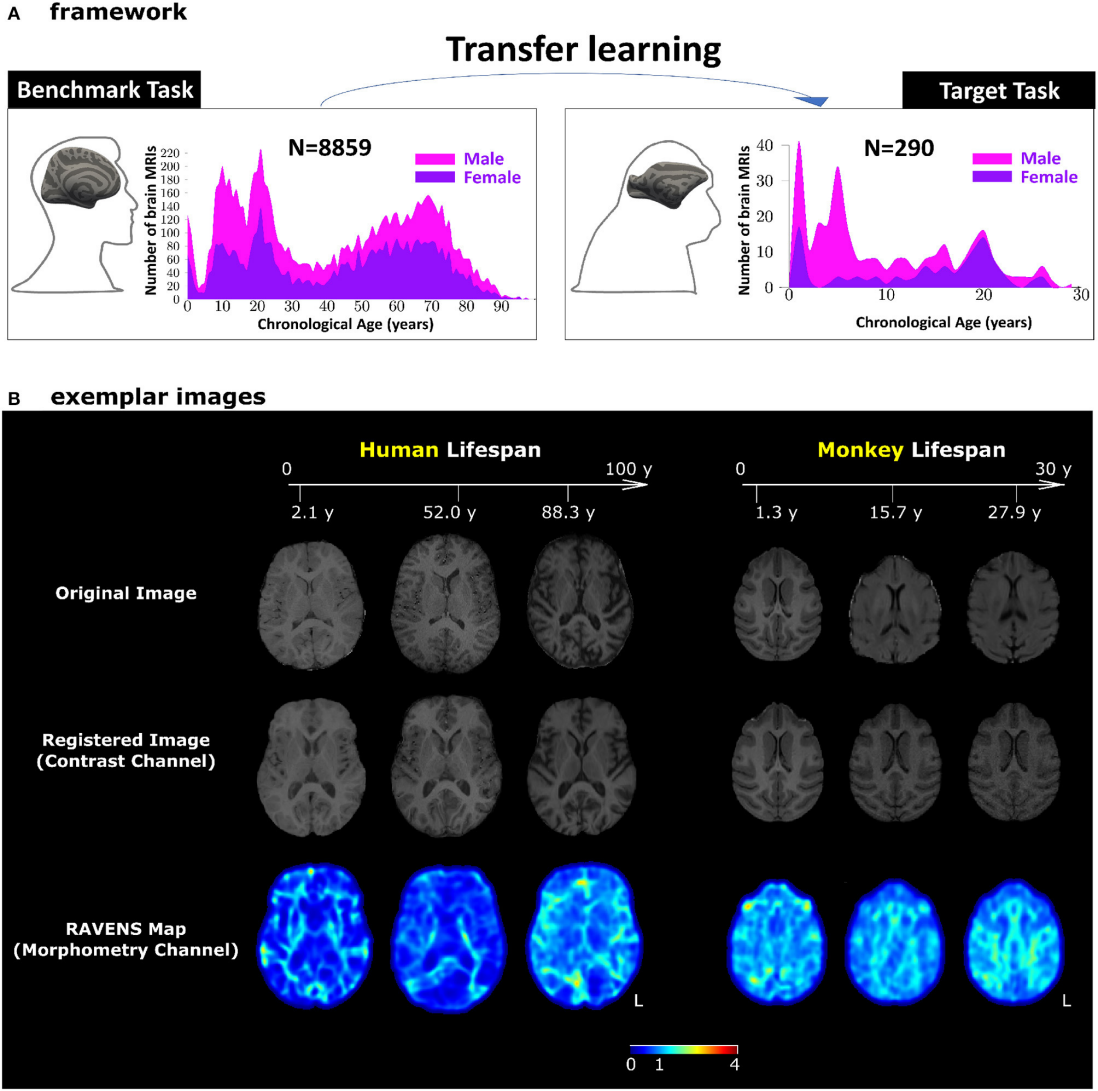
Our transfer learning framework. **(A)** Pre-trained AI models for human brain age estimation (benchmark task, left panel) were transferred to facilitate monkey brain age estimation (target task, right panel). In each panel, the histograms show sample sizes and age distributions of the study population. **(B)** Exemplar images for human and monkey T1-weighted brain MRIs. The skull-stripped images in the first row had been split into two channels – registered images (second row) for the contrast channel and RAVENS tissue density maps (third row) for the morphometry channel. Both channels were in the species-specific template space. The values in the RAVENS maps, as coded in the color bar, quantified the volumetric ratio (< , =, and >1 for volumetric shrinkage, preservation, and expansion) in the subject as compared to the template at each voxel.

For human brain T1-weighted brain MRI, the pre-processing included N4 bias correction (Tustison et al., 2010), field of view normalization (Ou et al., 2018), multi-atlas skull stripping (MASS) (Doshi et al., 2013; Ou et al., 2015), and a deformable registration of the skull-stripped image into the SRI24 atlas space (Rohlfing et al., 2010) by the deformable registration via attribute matching and mutual-saliency weighting (DRAMMS) algorithm (Ou et al., 2011, 2014). The deformable registration split the T1-weighted human brain MRI into two channels, both in the atlas space: a registered intensity image channel for the **contrast** information and a tissue density map known as RAVENS map (regional analysis of volumes examined in normalized space) containing **morphometry** information (Davatzikos et al., 2001). See Figure 1B for these two channels for exemplar subjects. Our previous study demonstrated that splitting a single T1-weighted MRI into these two channels led to a higher brain age estimation accuracy (He et al., 2021b, 2022). Therefore, this study used both channels for age estimation.

Monkey T1-weighted brain MRIs underwent the AFNI pipeline (animalwarper tool) to generate skull-stripped images (Saad et al., 2009; Jung et al., 2021), by non-rigidly transforming NIMH Macaque Template (NMT v2) into each monkey's brain MRI space. The skull-stripped monkey brain MRI in the native space then went through DRAMMS deformable registration to be spatially normalized into the NMT atlas space. This split the monkey brain MRI into a registered intensity image (i.e., the **contrast** channel) and the RAVENS map (i.e., the **morphometry** channel) similar to those in human brain MRIs. The right panel of Figure 1B shows exemplar monkey subjects for their images and the two channels we used for age estimation.

After cropping unused background in the pre-processed images, the final dimensions were 100 × 128 × 156 for monkey images in the NMT atlas space and 120 × 130 × 170 for brain images in the SRI24 atlas space. For the purpose of transfer learning, further resizing was done to linearly scale (not transforming) the human brain images to have the same size as monkey brains.

To train the deep learning model, the intensity map and RAVENS map are concatenated as one input image with two channels, with the size of 2 × 100 × 128 × 156.

### Five AI models

Five different DL AI models were evaluated for monkey brain age estimation. They included the general 2D/3D ResNet models (He et al., 2016), which were not specifically designed for but have been previously validated for brain age estimation (He et al., 2021a, 2022). We also included three models that were specifically designed for brain age estimation: 3D simple fully convolutional network (SFCN) (Peng et al., 2021), 2D global-local transformer (GL-Transformer) (He et al., 2021a), and 3D deep relation transformer (Relation-Transformer) (He et al., 2022). For the 2D models, we consider the 3D MRIs as a stack of 2D slices with different channels.

We chose these five AI models for their representativeness. On one note, these five models represent 2D and 3D typical deep learning architectures. The 2D ResNet and GL-Transformer are 2D deep learning architectures. The 3D ResNet, SFCN, and Relation-Transformer are 3D deep learning architectures. Moreover, these five models represent both the convolutional neural network (CNN) and transformer-based algorithms. The 2D/3D ResNet and SFCN are CNN-based, whereas the GL-Transformer and Relation-Transformer are based on transformers, which are recently popular deep learning architectures based on attention mechanisms (Vaswani et al., 2017; He et al., 2022). Loosely speaking, CNN uses voxel/pixel arrays, whereas transformer models split the images into visual tokens. They are two main screams of deep learning models for image computing.

The algorithms behind these models are briefly described below. The ResNet model contains residual block with skip connection to reuse the feature feeding into the residual block. The SFCN is a short version of the VGGnet (Simonyan and Zisserman, 2014), with only five convolutional layers to extract deep features. Global-Local transformer contains a transformer (Vaswani et al., 2017) block to optimal fuse global context and local information from patches to explore more detailed brain age information on brain MRIs. Relation-Transformer (He et al., 2022) uses the attention mechanism to learn four relations, such as the cumulative, relative, maximal, and minimal relations from a pair of input images.

### Training from scratch in monkey brain MRIs

The models trained from scratch on monkey MRIs used the same training configurations as in human MRIs: batch size was 8, the initial learning rate of the Adam optimizer (Kingma and Ba, 2014) was 0.0001 and was reduced to half at every 50 epochs with a total of 200 training epochs.

### Transferring and refining pre-trained models from human to monkey MRIs

Pre-training was done in human brain MRIs. All five models were trained with the mean absolute error (MAE) as the loss function, the same as in other human brain age estimation studies (Bashyam et al., 2020; Feng et al., 2020; He et al., 2021a,b, 2022; Peng et al., 2021). Parameters on models were optimized using the Adam optimizer (Kingma and Ba, 2014), with an initial learning rate of 0.001, which was reduced to half at every 20 epochs with a total of 80 training epochs. The batch size was set to 16. All other parameters were set to default provided by the PyTorch platform, similar to existing studies (He et al., 2021a,b, 2022).

Transfer learning was done using the pre-trained models from human brain MRIs as an initialization. The refinement was done in two stages. In Stage 1, we refined the last fully connected layer from scratch for monkey age estimation. We fixed the parameters on convolutional layers for feature extraction. This was done with 100 epochs on monkey brain MRIs with a learning rate of 0.001. After that, Stage 2 fine-tuned the parameters in all layers of the deep learning AI models with a learning rate of 0.0005, which was quartered at the 100^*th*^ epoch. All models were trained with 200 epochs. Although the age ranges of human and monkey are not the same, age matching is not necessary for transfer learning since the last fully connected layer is randomly initialized for monkey and fine-tuned in transfer learning.

### Ranking AI strategies by accuracy and stability

Ten AI strategies (five AI models, each with or without transfer learning) were compared.

**Accuracy**. We used a 10-fold cross-validation to measure the accuracy. Given *N* monkey brain MRIs, we randomly split them into 10 equal numbers of non-overlapping samples. Each time, one fold was selected for testing, and the rest were used for training. One metric for accuracy was the mean absolute error (MAE), $\text{MAE}=\frac{1}{N}\overset{N}{\underset{i}{\sum }}|{ŷ}_{i}-y_{i}|$, where ŷ is the AI-estimated age, while *y* is the chronological age. Smaller MAE corresponds to higher accuracy. The second accuracy metric we used was Spearman's correlation coefficient between the chronological and AI-estimated monkey brain ages. Spearman's correlation coefficient ranges from 0 (lowest accuracy) to 1 (highest accuracy). In addition to reporting the overall accuracy across the lifespan in the main text, we also computed the accuracy metrics in different age groups in Supplementary material. A monkey's lifespan capacity is approximately 0–35 years, with a rough ratio of 1:3 to human lifespan ages (Tigges et al., 1988). Therefore, we defined and compared three age groups within the current cohort, as previously defined (Simmons, 2016): young monkeys (age≤5, *n* = 88), middle-aged (5 <age< 20, *n* = 157), aged (age≥ 20, *n* = 45).

**Stability**. Each AI strategy was applied to varying sample sizes in our monkey brain MRI dataset. A strategy is more stable if the accuracy dropped the least going from using all 290 monkey brain MRIs to using only 10, 20, 30, …, and 90% of them. Training samples were randomly selected on each fold (10-fold cross-validation). For example, when sampling 20% for training, we randomly selected 20% samples from the 9-fold for training and the rest fold for testing and repeat 10 times.

**Ranking by accuracy and stability**. We ranked the 10 AI strategies from 1 (least preferable) to 10 (most preferable) in each of the four accuracy metrics and three stability metrics. The total rank score is a summation of those 7 scores ranging from 7 (least preferable) to 70 (most preferable). The four accuracy metrics were MAE ranked from highest (rank score 1) to the lowest (rank score 10) when using 100% of the monkey brain MRIs; Spearman's ρ ranked from the lowest (rank score 1) to the highest (rank score 10) when using 100% of the monkey brain MRIs; MAE ranked from highest (rank score 1) to the lowest (rank score 10) when using 30% of the monkey brain MRIs; and Spearman's ρ ranked from the lowest (rank score 1) to the highest (rank score 10) when using 30% of the monkey brain MRIs. The three stability metrics were the increase of MAE ranked from the highest (rank score 1) to the lowest (rank score 10) from using 100% to 30% of the monkey brain MRIs, the increase of MAE ranked from the highest (rank score 1) to the lowest (rank score 10) from using 100% to 20% of the monkey brain MRIs, and the increase of MAE ranked from the highest (rank score 1) to the lowest (rank score 10) from using 100% to 10% of the monkey brain MRIs. The AI strategies with the highest ranking scores (ideally close to 70) were recommended among the 10 strategies.

### Interpretation

For further interpretation of the brain age models, saliency maps were generated to show the most informative voxels from MRI scans selected by the AI model for brain age estimation. In brief, the integrated gradients were used (Sundararajan et al., 2017) to visualize the relationship between input images and predictions. The baseline was the image with zero values and a sequence of images was generated by a liner interpolation between the baseline and input image, yielding a sequence of output prediction and a sequence of gradient maps on input images.

The saliency maps shown in **Figure 4** were computed as the average saliency maps across all subjects in corresponding age groups (<5 and ≥5 years for monkeys and <15 and ≥15 for humans). To quantify the tissue-specific and regional contribution to brain age estimations, we applied lobular segmentations for gray and white matter regions, as well as the inclusion of the subcortical structure as a separate region. Mean saliency values normalized by whole sample minimum and maximum saliency values for each age group were quantified for tissue-based regional segmentation and plotted to visualize the trajectories of mean saliency.

Topographical saliency maps are prepared for two age groups for each channel used in AI models (intensity/contrast & RAVENS/morphometry) and captured from three different axes.

Similar procedures were carried out to generate human saliency maps and quantified saliency trajectories in matching age groups (under 15 years old and 15 or older) (**Figure 4**).

## Results

### Setup of transfer learning

The benchmark task was human brain age estimation (Figure 1A, left panel). It was based on using 8,859 T1-weighted human brain MRIs 0-97 years of age. The target task was monkey brain age estimation (Figure 1A, right panel), using T1-weighted monkey brain MRI from 290 monkeys with a lifespan of 1–30 years of age. We applied minimum pre-processing to split each bias-corrected and skull-stripped T1-weighted MRI into a 3D intensity image (for contrast information) and a 3D RAVENS (regional analysis of volumes examined in normalized space) map (for morphometry information) in the human or monkey atlas spaces (Seidlitz et al., 2018; He et al., 2021a,b, 2022; Jung et al., 2021), as shown in Figure 1B. Age estimation in this study, with or without transfer learning, was based on concatenating and using these two channels of images.

The five AI models we used in the study included two 2D AI models [the classic 2D ResNet He et al., 2016, our 2D global-local transformer (GL-Transformer) He et al., 2021a] and three 3D AI models (the classic 3D ResNet He et al., 2016, a recently-developed 3D SFCN Peng et al., 2021, and our 3D Relation Transformer He et al., 2022). These five models also include three convolutional neural network models (2D/3D ResNet, 3D SFCN) and two transformer models, that are the two main branches of deep learning AI architectures.

### Transfer learning improved accuracy, especially in very small sample sizes

**Figure 2:**
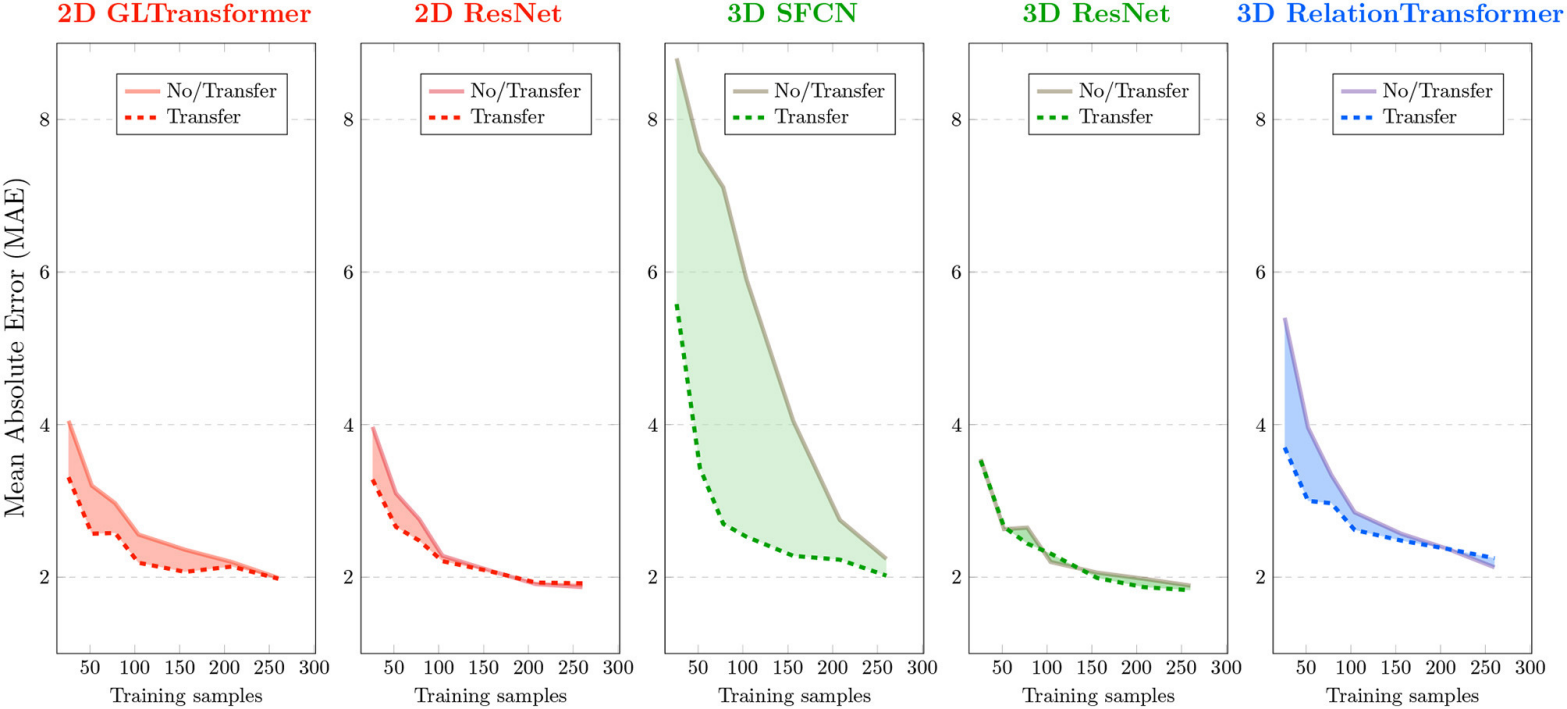
Effects of transfer learning in monkey brain age estimation. Each panel corresponds to an AI model that was used in this study. Brain age errors (mean absolute errors or MAEs) in 10-fold cross-validation of monkey brains are plotted on the y-axis, and various sample sizes used for AI are plotted on the x-axis. Solid curves are errors without transfer (i.e., training from monkey brain MRIs from scratch). Dashed curves are errors with transfer (from human to monkey). Red colors are used for 2D AI models, green for 3D AI models, and blue for a special 3D AI model that uses *N*^2^ input image pairs given *N* input images.

We performed transfer learning by first fixing the parameters from the pre-trained benchmark (human) model on convolutional layers, to refine the last fully-connected layer from scratch, and then performed refinement of all the parameters during target (monkey) model training. Figure 2 shows that transferring AI models from human to monkey brain MRIs (dashed curves) led to reduced age estimation errors (y-axis) for all five AI models compared to training directly in monkey data from scratch (solid curves). The effect varied, though, by sample size in the target task (x-axis) and across models.

The effect of transfer learning on AI accuracy was more pronounced when we used only <150, especially <50 (*p* < 0.05 for t-Test), randomly-sampled monkey MRIs. The benefit of transfer learning became smaller or even vanished for some AI models when using more than 200 randomly-sampled monkey MRIs. This general trend is consistent with all five models we tested although the specific cutoff sample size varied for different underlying AI models, as described below.

### Effects of transfer varied by AI models

We evaluated the performances of the AI models using mean absolute error (MAE) and Spearman's correlation coefficient (ρ) between the actual and the AI-estimated ages. A lower MAE and a higher ρ usually correspond to higher accuracy. The lowest MAE in the analysis of the 290 monkey brain MRIs occurred when using 3D ResNet with transfer learning (MAE=1.83 ± 0.39 years). The highest Pearson correlation ρ occurred when using the 3D SFCN with transfer learning (ρ = 0.921).

Two 3D AI models (3D SFCN and 3D relation transformer) benefited more from transfer learning than the two 2D AI models (2D ResNet and 2D GL-Transformer). In general, 3D AI models often required larger sample sizes than 2D models (Schulz et al., 2020).

The AI model that benefited the most was the model with the largest errors (3D SFCN). AI models that had a relatively smaller error (<4 years), even with just 30 randomly-sampled monkey brain MRIs, benefited less from transfer learning.

Of special note is the 3D relation transformer, which, given *N* input images, used *N*^2^ pairs of images as training samples. The 3D relation transformer estimated the relationship (sum, difference, bigger/smaller than) on every pair of input images (He et al., 2022). Therefore, transfer learning reduced errors compared to without transfer learning for the 3D relation transformer, with 220 or fewer input images (<48,400 input image pairs). The MAE of transfer learning became larger than the MAE without transfer learning with more than 220 input monkey brain MRIs (more than 48,400 input MRI pairs).

In general, the comparison across the underlying AI models showed that those AI models that needed more input MRIs (e.g., 3D SFCN and 3D ResNet) would benefit more from transfer learning.

### Transfer learning also improved stability

**Figure 3:**
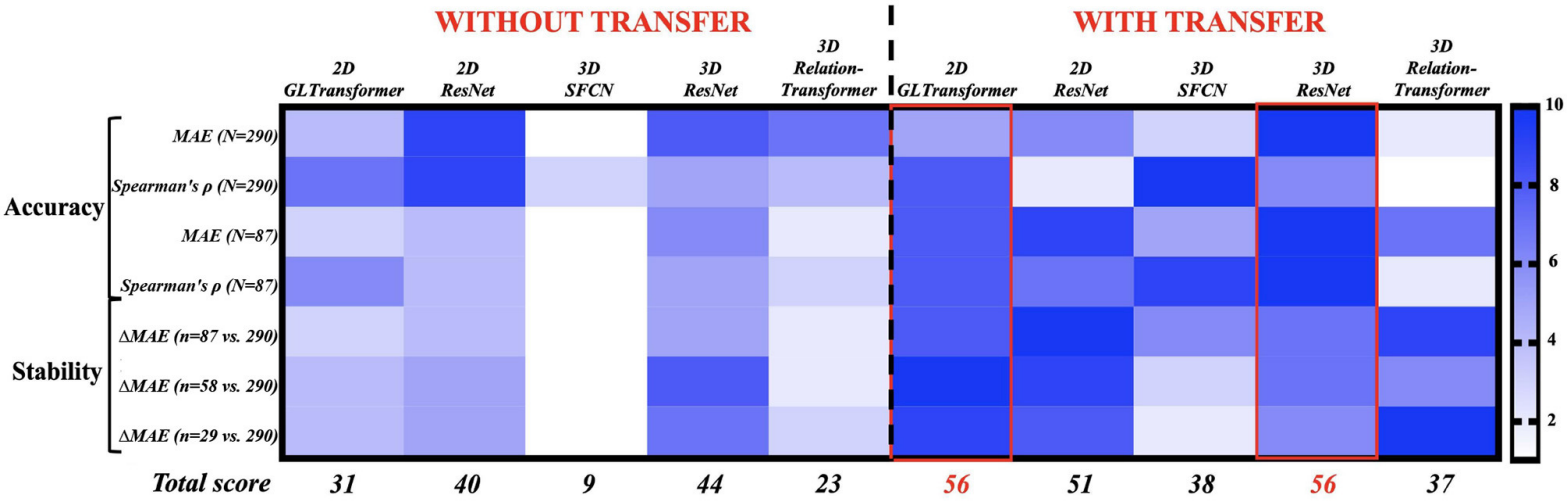
Ranking 10 AI strategies by accuracy (Rows 1–4) and stability (Rows 5–7). Each row assigns 10 AI strategies a rank score of 1 (least preferable, light blue) to 10 (most preferable, dark blue). The AI strategies with the highest total rank scores **(bottom row)** are the most accurate and stable choices for monkey brain age estimation. The exact values in those 10 accuracy and stability metrics can be found in Figure 2 and Supplementary Table S1.

We next ranked the 10 strategies (five AI models; with or without transfer learning) based on their accuracy and stability (Figure 3). The rank score ranged from 1 (least recommended) to 10 (most recommended) in four accuracy metrics and three stability metrics (with each row being a metric). Accuracy was measured as the mean absolute errors (MAE, smaller is better) and Spearman's correlation (higher is better) at 30% and 100% of the samples between AI-estimated and actual chronological monkey ages. Stability was measured by the increase of MAE (smaller is better) from when using 100% of the samples to when using 10%, 20%, and 30% of the samples. Overall, the 2D GL-Transformer with transfer learning and the 3D ResNet with transfer learning received the highest total rank scores, both scored 56 out of 70. They were followed by 2D ResNet with transfer learning, with a total rank score of 51. These recommended choices were all with transfer learning.

### Neuroanatomical interpretations

**Figure 4:**
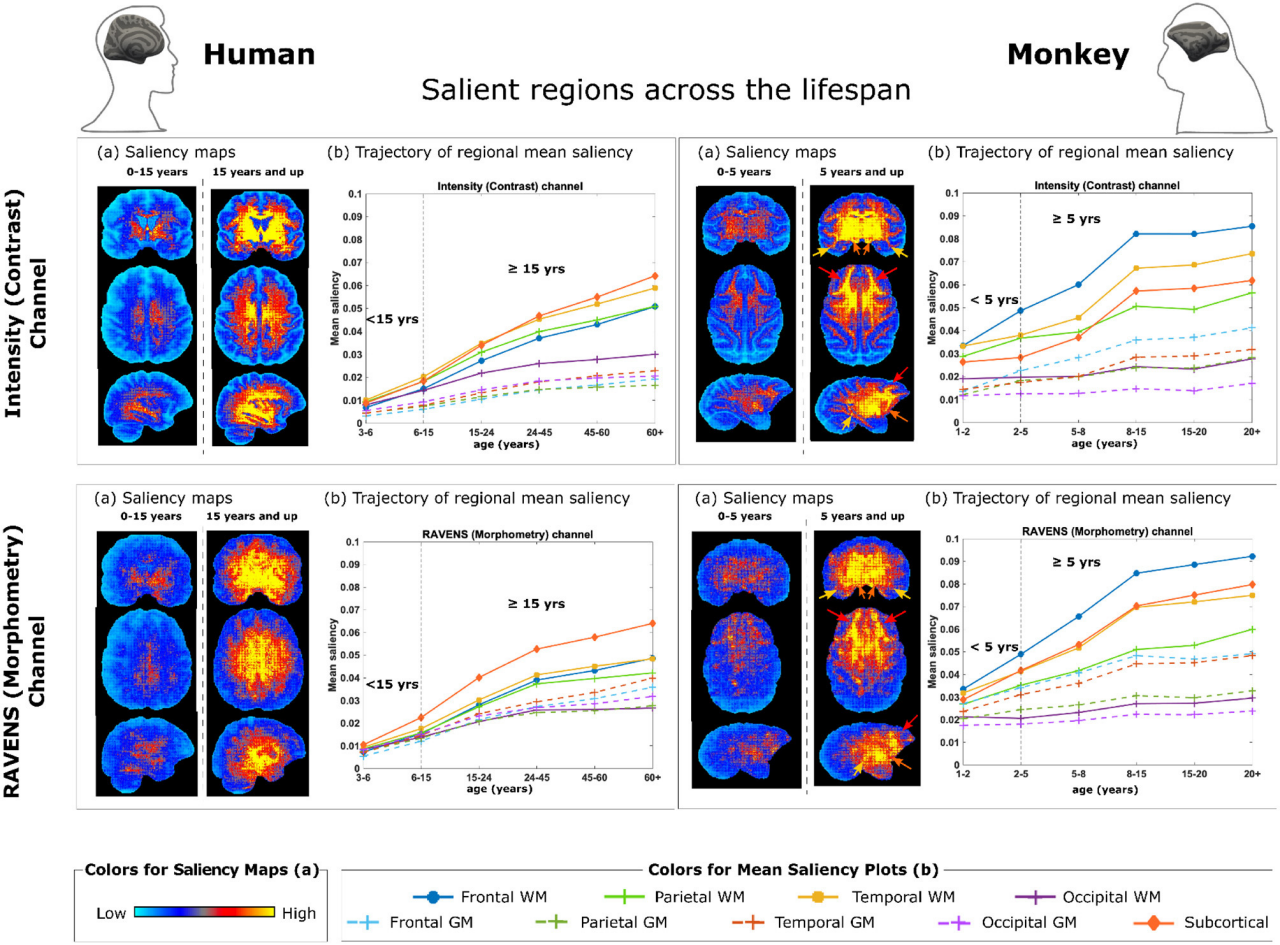
Brain regions and metrics contributing to monkey brain age estimation by 3D ResNet with transfer learning. (a) Saliency maps quantifying voxel-wise contribution in the intensity (contrast) channel **(Top)** and in the RAVENS (morphometry) channel **(Bottom)**, with humans on the left and monkeys on the right. Each saliency map is an average of an individual's saliency maps across all individuals in the corresponding age group. (b) Regions contribute the most to age estimation, by taking the mean saliency values in corresponding age groups in each brain region. The arrows in monkey saliency maps in panels (a) point out key regions demonstrated in panels (b): red - frontal white matter (WM), orange—temporal WM, and yellow—subcortical structures (including basal ganglia, diencephalon, brain stem, and cerebellum).

After examining the accuracy and stability of different AI models, we visualized the key MRI regions and features (from the contrast and morphometry channels) that contributed the most to age estimation in the best-performing model, 3D ResNet model with transfer learning, which had the highest rank scores in Figure 3 and the lowest MAE when all 290 MRIs were used in cross-validation. We generated saliency maps, which are topographical representations of feature importance, showing the most informative voxels selected by the AI model for brain age estimation (Figure 4). Three main observations are noted when comparing saliency maps generated from the pre-trained human model:

- For rhesus monkeys 5 years of age or younger, the salient regions selected by the AI model were more global throughout the brain. This can be seen in the right panels in Figure 4(a). Quantitative results in 1-5 years in Figure 4(b) also confirmed this observation.
- For rhesus monkeys 5 years of age and older, the aging effect became more localized in specific regions or tissue types. Compared to monkeys younger than 5 years of age, AI estimated ages for monkeys 5 years and older was higher/more reliant in the frontal white matter [pointed out by red arrows in Figure 4 (a, left)], temporal white matter (orange arrows) and subcortical structures (yellow arrows, including basal ganglia, diencephalon, brain stem, and cerebellum), followed distantly by the parietal white matter. The age information came from both the contrast (intensity) and the morphometry (RAVENS) channels. This can be visualized under monkey panels in Figure 4(a), as well as quantified in Figure 4(b) for 5 years and up.
- A similar trend is observed in humans under the age of 15 where salient regions are globally distributed and mean saliency values are comparable across regions [Figure 4 (left)], whereas in humans 15 years old and older (matching to 5 years old and older in monkeys), subcortical structures were the most prominent, followed by temporal, frontal, and parietal white matters [Figure 4 (left)].

## Discussion

Following the advances in medicine and nutrition, the longer life expectancy worldwide led to a growing elderly population and an increased healthcare burden on neurodegenerative diseases. Rhesus monkeys are particularly important in mapping longitudinal normal and pathological aging trajectories because of their high genetic and physiological similarity to humans, which effectively helped to bridge the critical gap between rodent experiments and human trials for developing potential therapeutics (Gray and Barnes, 2019). However, the limited sample sizes of monkey studies present a major obstacle to the reproducibility and wider application of this model in biomedical research. As a potential solution, we built and improved AI models with transfer learning from a large-sample (human) benchmark task to small-sample (monkey) target tasks (Kermany et al., 2018) to study brain aging using human and monkey MRI scans. While existing studies primarily focused on transferring pre-trained 2D AI models to 2D images or 2D slices within 3D images (Kather et al., 2019; Bashyam et al., 2020; Hollon et al., 2020; Ström et al., 2020), recent advances in 3D AI models (He et al., 2016; Feng et al., 2020), including our models (He et al., 2021a,b, 2022), which have been pre-trained on thousands to tens of thousands of 3D images (Figure 1), have created the opportunity to transfer pre-trained 3D AI models to 3D medical images. In the current study, we demonstrated that (1) 3D ResNet with transfer learning achieved the highest ranking in accuracy and stability out of 10 strategies (five AI models, with or without transfer), as well as the lowest errors when all 290 monkey MRIs were used (Figure 3), (2) transfer learning improved the accuracy and stability for monkey brain age estimation for all models (see the total ranking scores before versus after transfer in Figure 3), and the effect was more pronounced in very small sample sizes (especially for sample size ranges between 150 and 50, Figure 2), (3) the saliency maps generated by our best-performing model identified important brain age features in the frontal white matter, which is consistent with previous monkey studies (Wisco et al., 2008; Peters and Kemper, 2012). To the best of our knowledge, this is the first study that applied the DL framework combined with the transfer learning technique for brain age prediction using brain MRI from rhesus monkeys. Of note, there was one study that trained a non-deep-learning (DL) algorithm (relevance vector regression) on the tissue segmentations from 29 T1-weighted baboon brain MRIs for age estimation (Franke et al., 2017).

The current study sought to mitigate the small sample issue in building DL brain age models by applying transfer learning from pre-trained models from human to monkey. Indeed, we observed improved age prediction accuracy (indicated by the reduction of MAE in Figure 2) in all five models at various degrees. Interestingly, while we initially expected that more complex AI models [complexity is determined by the number of parameters and the floating point operations per second (flops)] may need more samples and thus would benefit more from transfer learning, our results did not conform with such an expectation. For instance, 3D SFCN [2.81 M(ega) parameters and 26.66 G(iga) flops] and 3D relation transformer (2.91 M parameters and 6.46 G flops) had fewer parameters than other AI models we used in this study but benefited the most from transfer learning. In contrast, 2D GL-Transformer (19.68 M parameters and 16.75 G flops), 2D ResNet (11.25 M parameters and 3.6 G flops), and 3 D ResNet (31.65 M parameters and 34.71 G flops)—three AI models with larger numbers of parameters (suggesting higher complexity)—actually benefited less from transfer learning. Instead of complexity as the major factor for determining the success of transfer learning, our results showed that AI models with larger errors at very small sample sizes (50 or under) actually benefited more from transfer learning. This suggests that, in addition to model complexity, sample sizes might be a key factor to consider when selecting the appropriate AI model for different tasks.

After evaluating the performances (based on accuracy and stability) of our DL models, we showed that the two top-ranked models (see Figure 3) are 3D ResNet with transfer learning (MAE = 1.83 years) and 2D GL-Transformer with transfer learning (MAE = 1.99 years). For brain age models, improved prediction accuracy may suggest a potentially higher sensitivity to identify subtle deviations from normal brain aging curves. In human, AI-estimated ages have identified accelerated aging for cognitive impairment (Liem et al., 2017; Poddar et al., 2019), traumatic brain injuries (Cole et al., 2015), schizophrenia (Cole et al., 2018), Alzheimer's disease (Franke et al., 2010), diabetes (Franke et al., 2013; Guan et al., 2022), smoking or alcohol use (Guggenmos et al., 2017; Ning et al., 2020), and early signs of future psychosis (Chung et al., 2018). Similarly, AI quantified the delayed human aging in long-term meditation practice (Luders et al., 2016), music-making (Rogenmoser et al., 2018), and higher education (Steffener et al., 2016). In these studies, the AI age estimation errors on human brain MRIs are around 3-6 years (Goyal et al., 2019; Bashyam et al., 2020; Smith et al., 2020; He et al., 2021b). With improvements in prediction accuracy and stability from transfer learning for monkey brain age estimation, it becomes possible to better quantify how diseases, genetics, environment, lifestyle, and other epigenetic factors have shifted monkey brains from normal aging curves (Jeon et al., 2018; Kuchan et al., 2020; Souder et al., 2021). This is especially useful because many of these factors can be well controlled in the laboratories for monkeys and other animal models, providing neuroscience testbeds that are otherwise difficult to obtain in human brains (Moss et al., 2007).

The current study also sought to improve the interpretability of the DL models and pinpoint the most important MRI features used by the best-performing models for age predictions. To achieve this, we generated saliency maps and showed that the highest-ranked AI strategy was able to learn monkey brain ages from both morphometry (RAVENS map) and the contrast (intensity image) information (Figure 4). The aging information was more widespread throughout the whole brain in monkeys under the age of 5 years but became progressively more localized for monkeys 5 years and more, which was also observed in the age-matched groups of humans (Figure 4). Interestingly, AI predicted monkey brain ages mostly based on MRI voxels located in the frontal white matter (WM) regions, followed by parietal WM and subcortical (mostly GM) regions (Figure 4). This agrees with previously established histological findings on monkey brain development across the lifespan, where WM degeneration was prevalent in aged animals (Peters, 2009; Peters and Kemper, 2012; Chen et al., 2013; Kubicki et al., 2019). These have mostly focused on the forebrain regions, showing mild dendritic and synaptic loss, and prominent WM degeneration in the dorsal prefrontal cortex, area 46, anterior cingulate cortex, and hippocampus during aging (Bowley et al., 2010; Luebke et al., 2010; Hara et al., 2012; Koo et al., 2012, 2013). We also observed that the rhesus monkey brain develops throughout young ages (under 5) (Malkova et al., 2006; Scott et al., 2016; Kim et al., 2020), which is similar to human brain development (Ou et al., 2017; Sotardi et al., 2021); WM undergoes more maturation and neurodegeneration similar to that in human; whereas the GM in monkey brain, unlike in human, experiences less to no significant changes in volume beyond very early maturation phases (Rakic et al., 1986; Ge et al., 2002; Peters and Kemper, 2012; Shi et al., 2013). The different feature salience from our model between humans and monkeys may be explained by neuroanatomical differences across species. For example, monkeys do not show increased neurofibrillary tangles and amyloid plaques, which are pathological hallmarks primarily affecting human hippocampal and parahippocampal regions during early stages of aging and dementia (Luebke et al., 2010). Moreover, evidence suggests functional specialization of the human brain for language related to the expansion of temporal WM connections, which may give rise to variation in WM connectome between humans and monkeys (Sierpowska et al., 2022). The differences in monkey salience features compared to those of humans may reflect that our pre-trained AI models were well-adapted to the animal dataset after transfer learning.

There are several limitations of the study that we plan to address in our future analyses. First, we observed a regression-to-the-mean (RTM) issue, similar to previous studies on age estimation in human brain MRIs (Bashyam et al., 2020; Feng et al., 2020; He et al., 2021b). Specifically, younger monkey brains were predicted older, and older brains were predicted younger (Supplementary Table S1 and Supplementary Figure S1). This is a well-known methodological issue for AI-based age estimation models. For our follow-up analyses, we aimed to address the RTM issue by re-balancing sample distribution across ages (Feng et al., 2020) or post-correction of AI-estimated brain ages (Liang et al., 2019; Peng et al., 2021). These procedures are ongoing work in the field (de Lange and Cole, 2020) but outside the main focus of the current study (transfer learning). Second, this study only focused on T1-weighted brain MRIs, similar to most AI brain age estimation articles in the human brain, as T1-weighted MRIs are most common in a large cohort (Bashyam et al., 2020; Feng et al., 2020; He et al., 2021b; Peng et al., 2021). Future studies may include diffusion and functional MRIs to further improve model performances. Third, among the 290 T1-weighted brain MRIs from 274 monkeys, there were 16 longitudinal scans or 6% of the monkeys had longitudinal scans, which is a much lower percentage of longitudinal scans compared to human brain age prediction studies with only dozen to lower hundreds of subjects (Zhang et al., 2018; Hu et al., 2019). It is possible that the addition of more longitudinal scans or using longitudinal information as a prior may improve model predictions. Fourth, we are yet to test the effect of scanner or site on AI accuracy in this study, similar to other human brain age estimation studies that also merged data from 10+ sites, and also due to heavily unbalanced data across sites (eight sites had less than five subjects, see Supplementary Table S2) and across scanners (95.5% of the scans were acquired on 3T scanners). Fifth, the saturated and highest possible accuracy in monkey age estimation remains unanswered due to the lack of large-scale monkey brain MRIs. It took at least 8,000 brain MRIs to saturate human brain age estimation (Kaufmann et al., 2019; Schulz et al., 2020). Nonetheless, we showed that given the limited monkey brain MRIs (dozens to lower hundreds), transfer learning brings higher accuracy and stability to AI models than without transfer learning. Our best-performing model also identified biologically meaningful age-related features and showed successful adaptation of the pre-trained model to the small sample target population as reflected by different saliency regions highlighted by the human pre-trained model and transferred monkey model which are consistent with previous studies.

Despite the limitations, we showed that transfer learning has a great potential to mitigate the small sample size problem in AI-driven brain age estimation using monkey MRIs and identified key features of aging in the frontal WM regions. Given the AI brain age estimator's wide success in human brain research, this first DL-based, transfer-learning-powered, lifespan-compatible, highly accurate, and publicly-released monkey brain age estimator could provide a simple but holistic measure of how brain changes in aging. Our study will also open wide opportunities to study monkey and other laboratory animal models under a variety of normal and pathological conditions, such as to help differentiate animals of the same chronological age but demonstrating varying levels of cognitive impairments.

## Data availability statement

The datasets presented in this study can be found in online repositories. The names of the repository/repositories and accession number(s) can be found within the Supplementary material.

## Ethics statement

The studies involving humans were approved by Boston Children's Hospital and Harvard Medical School. The studies were conducted in accordance with the local legislation and institutional requirements. Written informed consent for participation was not required from the participants or the participants' legal guardians/next of kin in accordance with the national legislation and institutional requirements. The animal study was approved by the Boston University Institutional Animal Care and Use Committee.

## Author contributions

Study design: SH, B-BK, and YO. Data analysis: SH, YG, CHC, B-BK, and YO. Manuscript writing: SH, YG, CHC, TM, JL, RK, DR, B-BK, and YO. All authors contributed to the article and approved the submitted version.

## Footnotes

^1^ https://fcon_1000.projects.nitrc.org/indi/PRIMEcollections.html

^2^ https://data.donders.ru.nl/?1

^3^ https://brain-development.org/ixi-dataset
