# Supplementary material for: Human-to-monkey transfer learning identifies the frontal white matter as a key determinant for predicting monkey brain age
Source: Front Aging Neurosci. 2023 Nov 1;15:1249415. doi: 10.3389/fnagi.2023.1249415 (PMC10646581; doi:10.3389/fnagi.2023.1249415)
Supplement: Supplementary file 1 [file Data_Sheet_1.PDF]

Supplementary Materials

Table S1. Accuracy (MAE and Spearman's ρ) without/with transfer learning by 5 AI models in different age groups. The age groups are defined based on previous literature [**?**]: Young (<5 years), Middle-aged ($5 - 20$ years), Aged (>20 years).

Model	Young (n=88)		Middle-aged (n=157)		Aged (n=45)		All, lifespan (n=290)	
	MAE	ρ	MAE	ρ	MAE	ρ	MAE	ρ
GLTransformer (2D)	0.88 / 0.78	0.85 / 0.84	1.96 / 2.05	0.82 / 0.82	4.28 / 4.07	0.14 / 0.18	1.99 / 1.98	0.93 / 0.93
ResNet (2D)	0.65 / 0.67	0.84 / 0.83	1.98 / 2.05	0.82 / 0.80	3.86 / 3.92	0.24 / 0.27	1.87 / 1.92	0.94 / 0.93
SFCN (3D)	0.48 / 0.55	0.86 / 0.84	2.21 / 2.14	0.83 / 0.82	5.81 / 4.49	-0.16 / 0.18	2.24 / 2.02	0.93 / 0.94
ResNet (3D)	0.66 / 0.64	0.84 / 0.86	2.10 / 2.01	0.81 / 0.82	3.54 / 3.50	0.28 / 0.29	1.89 / 1.83	0.93 / 0.93
Relation Transformer (3D)	0.67 / 0.58	0.82 / 0.80	2.04 / 2.32	0.82 / 0.79	4.05 / 3.89	0.08 / 0.07	1.94 / 2.03	0.93 / 0.93

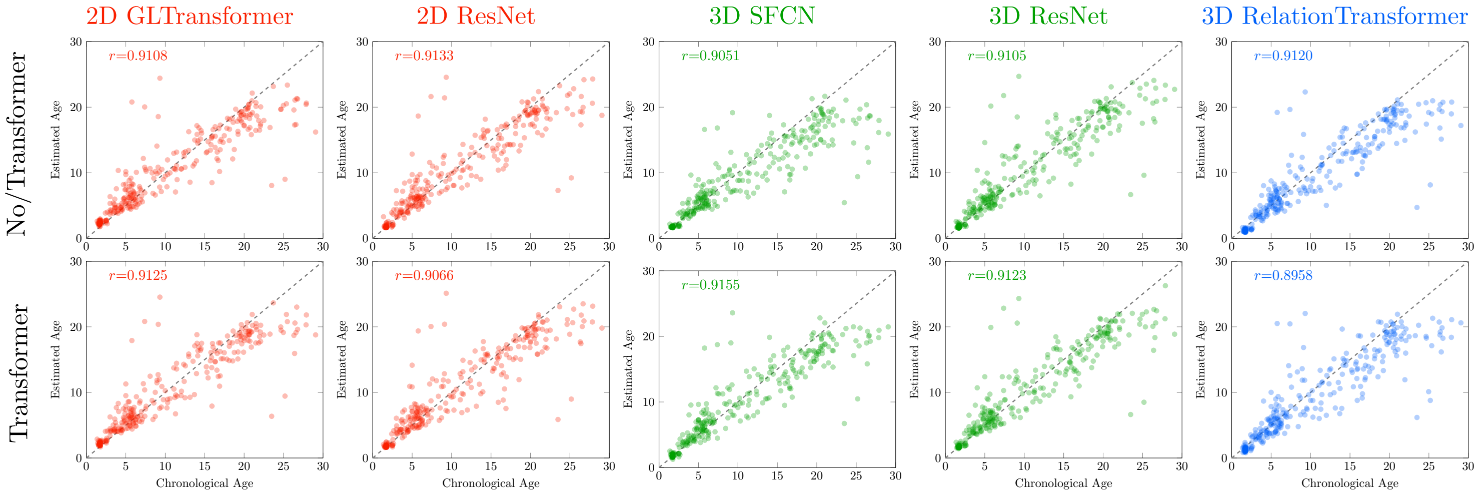

Fig. S1. The scatter plots of 5 AI models for age estimation after transfer learning, using all 290 monkey MRIs in 10-fold cross validation. r is the Pearson correlation between AI-estimated ages (y axes) and actual chronological ages (x axes). Red colors for 2D AI models, green for 3D AI models, and blue for special 3D AI models which used as input N^2 input image pairs given N input images.

Table S2. Monkey T1 structural MRI protocols.

Site	Scanner	Strength	Sequence	Resolution (mm)	TR/TE/TI (ms)	Flip Angle
Boston University (n=126+16longitudinal)	Philips Achieva (or Intera)	3T	3D-TFE	0.6/0.6/0.6	7/3/1000	8°
Aix-Marseille Université (n=4)	Siemens Prisma	3T	MPRAGE	0.8/0.8/0.8	2900/2.04/1000	8°
East China Normal University - Kwok (n=4)	Siemens Trio	3T	N/A	1.0/1.0/1.0	3000/77/1000	15°
Institute of Neuroscience (n=4)	Siemens Tim Trio	3T	MPRAGE	0.5/0.5/0.5	2500/3.12/1100	9°
McGill University (n=1)	Siemens Trio	3T	MP2RAGE	0.6/0.6/0.6	5000/3.65/2500	5°
Mount Sinai School of Medicine - Philips (n=8)	Philips Achieva	3T	N/A	0.5/0.5/0.5	1500/6.93/1100	8°
Mount Sinai School of Medicine - Siemens (n=5)	Siemens Skyra	3T	MPRAGE	0.5/0.5/0.5	4000/2.11/1820	8°
Newcastle University (n=10)	Siemens Skyra	4.7T	MDEFT	0.5/0.5/2.0	750/6/800	30°
Netherlands Institute for Neuroscience (n=1)	Philips Ingenia	3T	3D-FFE	0.6/0.6/0.6	13/6/900	8°
Nathan Kline Institute (n=2)	Siemens Tim Trio	3T	N/A	0.5/0.5/0.5	2500/3.87/1200	8°
Oregon Health and Science University (n=2)	Siemens Tim Trio	3T	Siemenstfi	1.5/1.5/1.5	2070/25/900	90°
University of Oxford (n=20)	N/A	3T	MPRAGE	0.5/0.5/0.5	2000/19/1100	8°
Princeton University NA&P Lab (n=2)	Siemens Prisma	3T	MPRAGE	0.5/0.5/0.5	2700/2.32/850	9°
Stem Cell and Brain Research Institute - 1.5T (n=13)	Siemens Sonata	1.5T	MPRAGE	0.6/0.6/0.6	2160/2890/1100	15°
Stem Cell and Brain Research Institute - 3T (n=3)	Siemens Prisma	3T	MPRAGE	0.5/0.5/0.5	3000/3.62/1100	8°
University of California Davis (n=19)	Siemens Skyra	3T	N/A	0.3/0.3/0.3	2500/3.65/1100	7°
University of Wisconsin-Madison (n=50)	GE Discovery	3T	N/A	0.27/0.5/0.27	211.4/5.412/600	10°
